# The Genetic Structure of Pacific Islanders

**DOI:** 10.1371/journal.pgen.0040019

**Published:** 2008-01-18

**Authors:** Jonathan S Friedlaender, Françoise R Friedlaender, Floyd A Reed, Kenneth K Kidd, Judith R Kidd, Geoffrey K Chambers, Rodney A Lea, Jun-Hun Loo, George Koki, Jason A Hodgson, D. Andrew Merriwether, James L Weber

**Affiliations:** 1 Anthropology Department, Temple University, Philadelphia, Pennsylvania, United States of America; 2 Independent Researcher, Philadelphia, Pennsylvania, United States of America; 3 Department of Biology, University of Maryland, College Park, Maryland, United States of America; 4 Department of Genetics, Yale University, New Haven, Connecticut, United States of America; 5 School of Biological Sciences, Victoria University, Wellington, New Zealand; 6 Transfusion Medicine Laboratory, Mackay Memorial Hospital, Taipei, Taiwan; 7 Institute for Medical Research, Goroka, Eastern Highlands Province, Papua New Guinea; 8 Department of Anthropology, Binghamton University, Binghamton, New York, United States of America; 9 Marshfield Clinic Research Foundation, Marshfield, Wisconsin, United States of America; University of Chicago, United States of America

## Abstract

Human genetic diversity in the Pacific has not been adequately sampled, particularly in Melanesia. As a result, population relationships there have been open to debate. A genome scan of autosomal markers (687 microsatellites and 203 insertions/deletions) on 952 individuals from 41 Pacific populations now provides the basis for understanding the remarkable nature of Melanesian variation, and for a more accurate comparison of these Pacific populations with previously studied groups from other regions. It also shows how textured human population variation can be in particular circumstances. Genetic diversity within individual Pacific populations is shown to be very low, while differentiation among Melanesian groups is high. Melanesian differentiation varies not only between islands, but also by island size and topographical complexity. The greatest distinctions are among the isolated groups in large island interiors, which are also the most internally homogeneous. The pattern loosely tracks language distinctions. Papuan-speaking groups are the most differentiated, and Austronesian or Oceanic-speaking groups, which tend to live along the coastlines, are more intermixed. A small “Austronesian” genetic signature (always <20%) was detected in less than half the Melanesian groups that speak Austronesian languages, and is entirely lacking in Papuan-speaking groups. Although the Polynesians are also distinctive, they tend to cluster with Micronesians, Taiwan Aborigines, and East Asians, and not Melanesians. These findings contribute to a resolution to the debates over Polynesian origins and their past interactions with Melanesians. With regard to genetics, the earlier studies had heavily relied on the evidence from single locus mitochondrial DNA or Y chromosome variation. Neither of these provided an unequivocal signal of phylogenetic relations or population intermixture proportions in the Pacific. Our analysis indicates the ancestors of Polynesians moved through Melanesia relatively rapidly and only intermixed to a very modest degree with the indigenous populations there.

## Author Summary

The origins and current genetic relationships of Pacific Islanders have been the subjects of interest and controversy for many decades. By analyzing the variation of a large number (687) of genetic markers in almost 1,000 individuals from 41 Pacific populations, and comparing these with East Asians and others, we contribute to the clarification and resolution of many of these issues. To judge by the populations in our survey, we find that Polynesians and Micronesians have almost no genetic relation to Melanesians, but instead are strongly related to East Asians, and particularly Taiwan Aborigines. A minority of Island Melanesian populations have indications of a small shared genetic ancestry with Polynesians and Micronesians (the ones that have this tie all speak related Austronesian languages). Inland groups who speak Papuan languages are particularly divergent and internally homogeneous. The genetic divergence among Island Melanesian populations, which is neatly organized by island, island size/topography, as well as their coastal or inland locations, is remarkable for such a small region, and enlarges our understanding of the texture of contemporary human variation.

## Introduction

**Figure 1:**
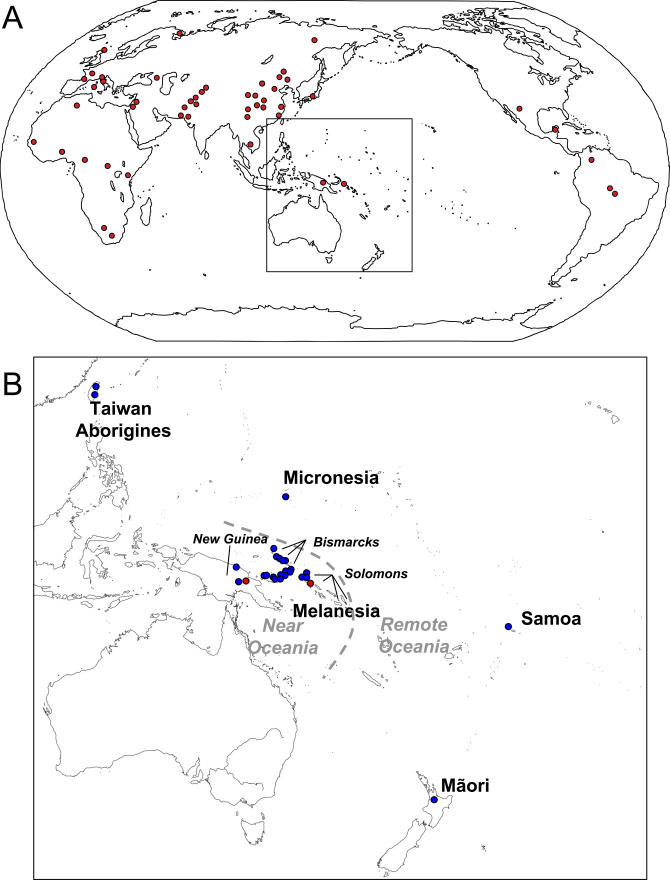
Populations Included in This Study (A) HGDP-CEPH population locations. The two Pacific groups are boxed. (B) Pacific population locations. Our population samples are blue; the 2 HGDP-CEPH Melanesian “Oceanic” groups are red.

The populations in New Guinea and the islands immediately to the east (the Bismarck and Solomons archipelagos) are well-known for their great diversity in cultures, languages, and genetics, which by a number of measures is unsurpassed for a region of this size [1]. This area is referred to as Near Oceania, as opposed to the islands farther out in the Pacific, known as Remote Oceania [2] (see Figure 1). For simplicity, we refer only to the peoples of Near Oceania as “Melanesians,” although this term ordinarily encompasses additional groups to the east as far as Fiji, who are not covered in this study. Major parts of Near Oceania were settled from Southeast Asia early in modern human prehistory, between ∼50,000 and ∼30,000 years before present (YBP) [3–5]. Populations were relatively isolated at this edge of the human species range for the following 25,000 years. The early settlers in Near Oceania were very small groups of hunter-gatherers. For example, New Ireland, which is more than 300 km long, is estimated to have had a pre-Neolithic carrying capacity of ∼1,200 people or fewer [6]. There is evidence of sporadic, modest contact between New Guinea and the Bismarcks from 22,000 YBP, and with Bougainville/Buka in the Solomons only from ∼3,300 years ago [3,7].

By ∼3,300 YBP [3], at least one powerful new impulse of influence had come from Austronesian speaking migrants from Island Southeast Asia, likely associated with the development of effective sailing [8], that led to the appearance of the Lapita Cultural Complex in the Bismarck Archipelago. After only a few hundred years, “Lapita People” from this area had colonized the islands in Remote Oceania as far east as Tonga and Samoa, where Polynesian culture then developed [9].

The distribution and relations of Pacific language families reflect ancient settlement. Austronesian is a widespread and clearly defined linguistic family with more than 1,000 member languages, which has its greatest diversity, and likely origin, in Taiwan ∼4,000–5,000 years ago [10]. Some basic phylogenetic relations within Austronesian are sketched in Figure S1. All Austronesian languages spoken outside Taiwan belong to the Malayo-Polynesian branch, and almost all the Malayo-Polynesian languages of Oceania belong to the Oceanic branch. It is Proto Oceanic, the immediate ancestor of the Oceanic languages, that is associated with an early phase of the Lapita Cultural Complex. Proto Oceanic split into a number of branches as its descendants spread across Remote Oceania, including Proto Nuclear Micronesian and Proto Polynesian (a branch of Central Oceanic).

Almost all the other indigenous languages of Oceania are referred to as non-Austronesian, or Papuan. Most Papuan languages are found in New Guinea, with the remainder in nearby islands. This is a residual category of ∼800 languages. Most of these can be assigned to more than 20 different language families, but these families cannot be shown to be related on present evidence. There remain a number of “Papuan” isolates that cannot be grouped at all [11]. Trans New Guinea is the largest Papuan language family. It consists of ∼400 languages and dates to 6,000 to 10,000 YBP [12]. Other Papuan families including the ones in the Bismarck and Solomon archipelagos probably also go back at least to this period [13–15]. While it is reasonable to assume these different Papuan families had common origins further back in time, any evidence of such ties that is recoverable with standard methods of historical linguistics has been erased over the millennia. The concentration and number of these apparently unrelated language families and isolates is unsurpassed in any other region of the world [15].

Analyses of genetic variation at some informative loci, particularly the mitochondrial DNA (mtDNA) (reviewed in [16,17–19]), non-recombining Y-chromosome markers (NRY) (reviewed in [19,20]), and a small set of autosomal microsatellites [21] have provided divergent impressions of the population genetic structure of both Near and Remote Oceania. Because they have ¼ the effective sample size of autosomal markers, the mtDNA and NRY haplotypes have been particularly subject to the effects of random genetic drift, and each autosomal marker, no matter how informative, still represents a minute fraction of the total genetic variation among populations. Even so, these data have shown that the genetic variation in Near Oceanic populations is considerably greater than in Remote Oceanic ones, and that there are a cluster of haplogroups that developed in particular islands of Near Oceania between approximately 50,000 and 30,000 years ago.

However, a number of unresolved issues remain concerning the proper interpretation of these and other data that a comprehensive genomic sampling of neutral biparental markers across Pacific populations should clarify. A list of these includes: 1) to whom are these diverse Melanesian populations most closely related outside this region (East or South Asians, or perhaps even Africans, whom they physically resemble)? 2) how does the genetic diversity and differentiation of Near Oceanic populations compare with those in other regions? 3) is there a clear organization of the variation among groups in Near Oceania (i.e., either by language, by island, or distance from major dispersal centers)? 4) is there a genetic signature of Aboriginal Taiwanese/Southeast Asian or Polynesian influence in Melanesian populations, especially in the Bismarcks, where the Lapita Cultural Complex developed? and 5) are Polynesians more closely related to Asian/Aboriginal Taiwanese populations or to Melanesians?

Here we report the analysis of 687 microsatellite and 203 insertion/deletion (indel) polymorphisms in 952 individuals from 41 Pacific populations, primarily in the Bismarck Archipelago and Bougainville Island, and also including select sample sets from New Guinea, Aboriginal Taiwan, Micronesia, and Polynesia. The results show the reduced internal variation of Near Oceanic Melanesian populations and the remarkable divergence among them, and how this divergence is influenced by island size and topography, and is also correlated with language affiliation. We also detected a very small but clear genetic signature of “Asian/Polynesian” intermixture in certain Austronesian (Oceanic)-speaking populations in the region (by “genetic signature,” we mean an ancestral proportion in some groups inferred by the STRUCTURE analysis that predominates in another ancestral grouping). For global context, these data were compared with data from the Centre d'Etude du Polymorphisme Humain human genome diversity panel (HGDP-CEPH), composed of cell lines [22–24], especially its subset from East Asia. Figure 1A shows how undersampled the Pacific populations had been in the HGDP-CEPH dataset (as well as its emphasis on particular regions of Asia), and Figure 1B shows the distribution of our Pacific population samples, with its intensive coverage in Near Oceania.

## Results

Our sampling strategy concentrated on Papuan-speaking populations and their immediate Oceanic-speaking neighbors from the islands immediately to the east of New Guinea, in what is called Northern Island Melanesia, consisting of the Bismarck and Solomon Archipelagos (see Figure 1B). The three largest islands of the region were most intensively sampled—New Britain, New Ireland, and Bougainville—along with two nearby smaller islands (New Hanover and Mussau). Additional Pacific samples came from New Guinea (one set from the lowland Sepik region and one set from the Eastern Highlands), Micronesia (primarily from Belau), Polynesia (Samoans and one New Zealand Mãori group), and aboriginal Taiwan (Amis and the Taroko, a mountain Atayal group). The details of the sample locations and language family affiliations are given in Table S1 and in the Methods section.

### The Global Context

**Figure 2:**
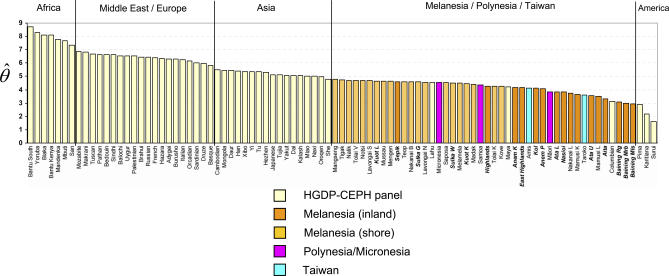
Population Diversity Values of *θ̂* for the HGDP-CEPH and Pacific datasets, for 687 microsatellites. Populations are ordered by their declining values of *θ̂*, but systematic regional distinctions are indicated by vertical lines. Conglomerate groups tend to have higher values than nearby populations (Bantu South, Sepik, Highlands, Micronesia, Samoa, and Columbia). Papuan-speaking groups are in bold italics; the Melanesian inland/shore distinction is indicated by the two shades of orange. Abbreviated names are spelled out in Table S1.


Figure 2 shows the estimated values of *θ* (*θ̂*) calculated from expected heterozygosity (*H_e_*) arranged from highest to lowest values, combining our Pacific populations and the HGDP-CEPH global set (the values of *θ̂*, *H_e_*, and the average number of alleles per locus are given in Table S1). From Ohta and Kimura [25], under a stepwise model, the expected relationship between *θ* and heterozygosity (*H*) is

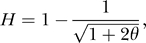


which rearranges to


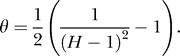


For autosomal loci, *θ* is defined as *θ* = 4*N_e_* *μ*, where *N_e_* is defined as the effective population size and *μ* is the per generation mutation rate. Assuming the mutation rate is constant across populations and that the stepwise mutation model is appropriate, *θ̂* provides an estimate that is linearly correlated with effective population size. In contrast, *H* asymptotically approaches a value of 1 as the effective population size increases. Therefore, the use of *θ̂* is more appropriate to represent differences in effective population sizes among populations (e.g., a *θ* ratio of 2 between two populations indicates twice the effective population size between the populations, while an *H* ratio of 2 does not).

The pattern of variation in Figure 2 is consistent with a series of successive founder effects that modern humans underwent in their expansions out of Africa (also shown by [26]). African populations have the highest values, followed in order by Europe/Central Asians, East Asians, Melanesians, and Native Americans. All the Pacific populations ranked together in a narrow band towards the low end of *θ̂* values (between 4.8 and 2.9). Within the Melanesian set, inland populations generally had lower values of *θ̂* than shore-dwelling groups, as shown. The three non-Pacific groups in the range between 4.8 and 2.9 were the Maya, Columbia, and Lahu. The Maya are known to have some European ancestry, which would explain their relatively high *θ̂* for a Native American group; and the Lahu are an Asian population that was subject to particularly strong random genetic drift [24]. Columbia and other conglomerate groups made up of individuals from different populations (e.g., Bantu South, Sepik, Highlands, Micronesia, and Samoa) consistently had higher values of *θ̂* than related groups. This combining of groups has caused inflated levels of diversity and effective population size estimates (i.e., there is more variation in a combined sample set than is typically contained in one from a clearly defined population).

**Figure 3:**
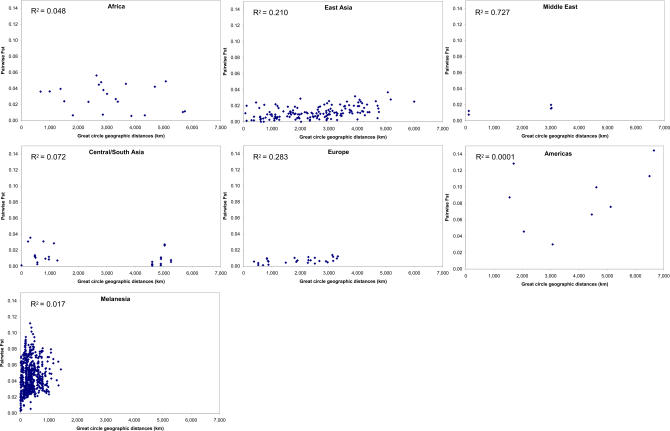
Genetic versus Geographic Distances within Continents Regional correlations between *F*_ST_ and geographic distance for population pairs.

Ramachandran et al. [26] investigated the correlation between geographic distance and genetic differentiation as measured by pairwise *F*_ST_ in the global HGDP-CEPH dataset, and found a linear relationship existed, with major deviations from the fitted line they believed consistent with admixture or extreme isolation. We analyzed this correlation by major region, adding our expanded Pacific dataset. The results, shown in Figure 3, show the extremely heterogeneous nature of the linear correlations and distributions from region to region. The sampled Melanesian populations were distributed across a comparatively small geographic area, but their range of pairwise *F*_ST_ values was extremely large. Only the Native American groups had an equivalent range of *F*_ST_ values, but these were unreliable since there were only five American populations distributed across very large distances.

To quantify the degree of variation within and among populations, an analysis of molecular variance (AMOVA) for the Pacific materials plus the HGDP-CEPH dataset was performed, with the results shown in Table 1. The global AMOVA results first presented in [24] for the HGDP-CEPH dataset were based on 377 microsatellites, included some first degree relatives, and included only two “Oceanic” populations (from the Nasioi of Bougainville and highland New Guinea). In the current analysis based on 687 microsatellites, the Americas had the highest among-population variation component, followed in order by Melanesia, Africa, Asia, and Europe. This pattern follows directly from their ranking in population heterozygosities or *θ̂* [27].

**Table 1:**
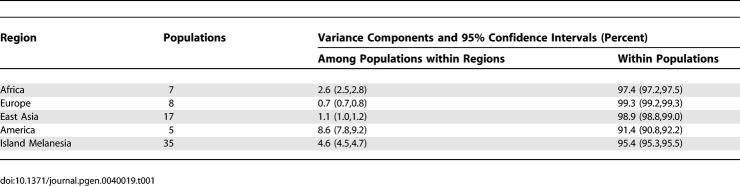
Analysis of Molecular Variance (AMOVA) for 687 Microsatellites for Major Regions (HGDP-CEPH plus Pacific)

As shown in Table 2, the microsatellite variation in Melanesia (New Guinea, New Britain, New Ireland, and Bougainville) was apportioned first by language group and then by island. While population variation among the different islands was considerable (refer to the 95% confidence interval), within-island variation among populations was more than three times greater. This was primarily due to the extensive variation within New Britain (with a 5% internal variance component), followed by Bougainville (3.7%), and New Ireland (2%, see Table S2). The variation among the three New Guinea samples in our series was lower, most likely because of their less rigorous population definitions (see the Methods section for sampling details).

**Table 2:**
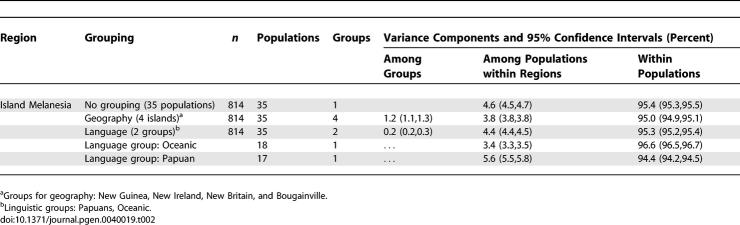
Analysis of Molecular Variance (AMOVA) for 687 Microsatellites for Island Melanesia (partitioned by Island and by Language Group)

Apportioning the molecular variance by language group (between Oceanic speaking and Papuan speaking populations) only accounted for 0.2 % of the total, which, as indicated by the very small 95% confidence interval, was still significant. Since the two language categories are scattered across the islands, geography and intermixture will confound possible language effects. While the microsatellite variation among the Oceanic-speaking populations was significant, it was much greater among the Papuan-speaking populations (many of which are located in the mountainous interiors of the larger islands).

To investigate individual and population similarities, we applied a Bayesian model-based clustering algorithm implemented in the STRUCTURE program [28] to our Pacific dataset combined with the HGDP-CEPH panel (also genotyped by the Marshfield Clinic). This program identifies groups of individuals who have similar allele frequency profiles. The great advantage of this clustering approach is that it avoids a priori population classifications, and instead estimates the shared population ancestry of individuals based solely on their genotypes under an assumption of Hardy-Weinberg equilibrium and linkage equilibrium in ancestral populations. It infers individual proportions of ancestry from *K* clusters, where *K* is specified in advance and corresponds to the number of posited ancestral populations; *K* can be varied across independent runs. Individuals can be assigned admixture estimates from multiple ancestral populations, with the admixture estimates summing to 1 across these population clusters.

**Figure 4:**
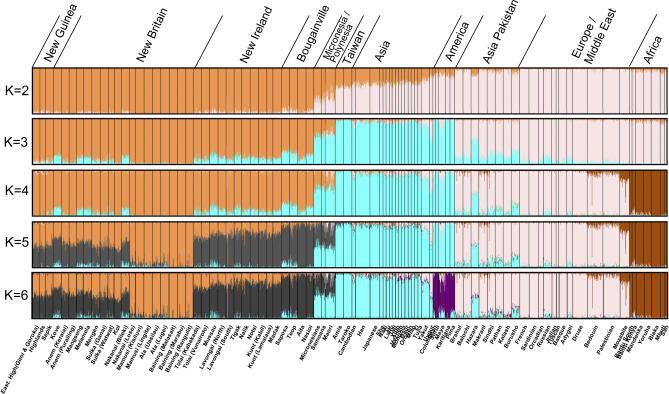
Global Population Structure STRUCTURE analysis of the Pacific and HGDP-CEPH sets combined, for 687 microsatellites and 203 indels over 91 populations encompassing 1,893 samples (20,000/10,000 burnin/MCMC). Each vertical line represents an individual. The colors represent the proportion of inferred ancestry from *K* ancestral populations.

Figure 4 presents the STRUCTURE analysis of our Pacific dataset plus the HGDP-CEPH Panel for 687 microsatellites and 203 indels on the 22 autosomes, on a total of 1,893 individuals from 91 populations. Each increase in *K* split a cluster that had been defined in an earlier run, and individuals from the same populations had very similar membership coefficients in the inferred clusters. Details of the STRUCTURE results are provided in the Table S3. Inclusion of our large Pacific dataset altered the sequence of splitting, but did not change, the five major global clusters that had previously identified with a smaller set of microsatellites: Sub-Saharan Africa, Western Eurasia, East Asia, “Oceania,” and the Americas [24]. The Taiwan Aborigines clustered with East Asia, while Polynesians and Micronesians had a mixed position between East Asians and Melanesians (“Oceania”). The Mãori had the suggestion of a minor proportion of European admixture, which had been indicated by the donors themselves.

There was a small but consistent “Asian/Polynesian” admixture estimate in specific Melanesian groups. Because clustering after *K* = 6 mostly involved Near Oceanic populations, we stopped the combined global analysis there, and analyzed the Pacific subset separately thereafter.

**Figure 5:**
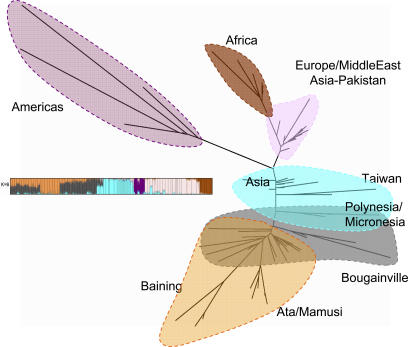
Global Population Tree Neighbor-joining *F*_ST_-based tree for the Pacific and HGDP-CEPH combined datasets (687 microsatellites). Superimposed colors are from the STRUCTURE analysis at *K* = 6 (also shown).

An unrooted neighbor-joining tree for the same HGDP-CEPH and Pacific samples, excluding the indels, was calculated from a matrix of pairwise *F*_ST_ “coancestry” distances (similar to Reynolds' D [29], see Table S4), and is shown in Figure 5. For comparison, the cluster colors for the *K = 6* STRUCTURE run were superimposed on the tree. The results were compatible with the clusters identified with STRUCTURE. Branch lengths varied inversely with values of *θ̂* or expected heterozygosity, so that populations with the longest branch lengths had the lowest values of *θ̂*. The longest branches belonged to the Native American and separate Melanesian groups. As with the STRUCTURE results, this unrooted *F*_ST_ based tree had Melanesians, East Asians, and Native Americans at the opposite end of the human tree from Africans and Europeans. Trees based on other population pairwise genetic distance matrices (Nei's chord distance [30], (δ*μ*)^2^ [31], the proportion of shared alleles [32], and Cavalli-Sforza and Edwards' chord distance [33]) also indicated relatively large distances between Africans and Melanesians, and also consistently placed the Taiwan Aborigines between the East Asians and Polynesians/Micronesians (Figure S2).

### The Pacific

**Figure 6:**
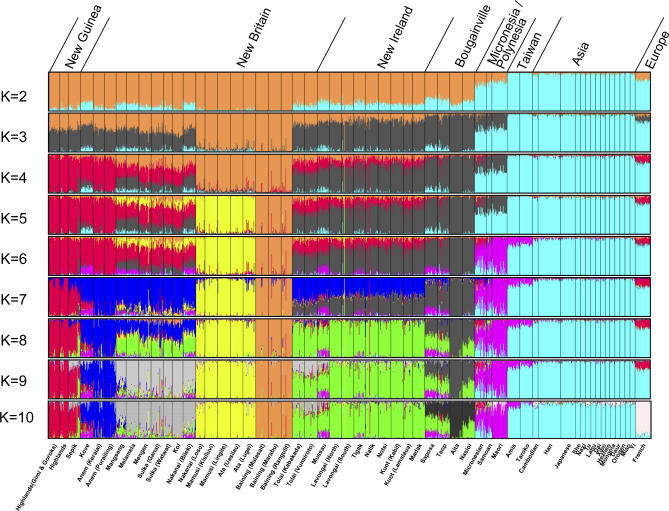
Pacific Population Structure STRUCTURE analysis of the Pacific, HGDP-CEPH East Asia, and European (French) groups (687 microsatellites and 203 indels, 20,000/10,000 burnin/MCMC). Results are given from *K* = 2 to *K* = 10. Each vertical line represents an individual. The colors represent the proportion of inferred ancestry from *K* ancestral populations.

**Figure 7:**
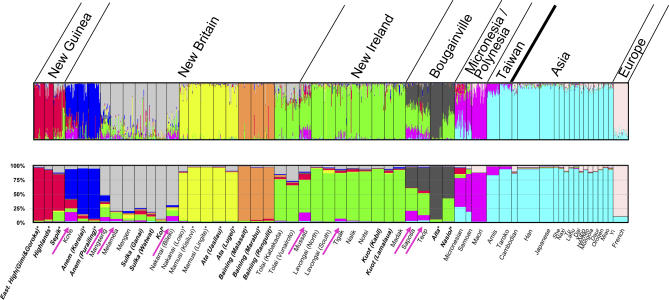
Pacific Population Structure Details Individual and (below) mean population assignments at *K* = 10 for the Pacific, HGDP-CEPH East Asia, and French. Purple arrows denote the eight Oceanic-speaking populations with an “Austronesian” assignment signature above 5%. Papuan-speaking group names are in bold italics. Asterisks denote inland groups. Populations are arranged geographically, approximately from west to east.

**Figure 8:**
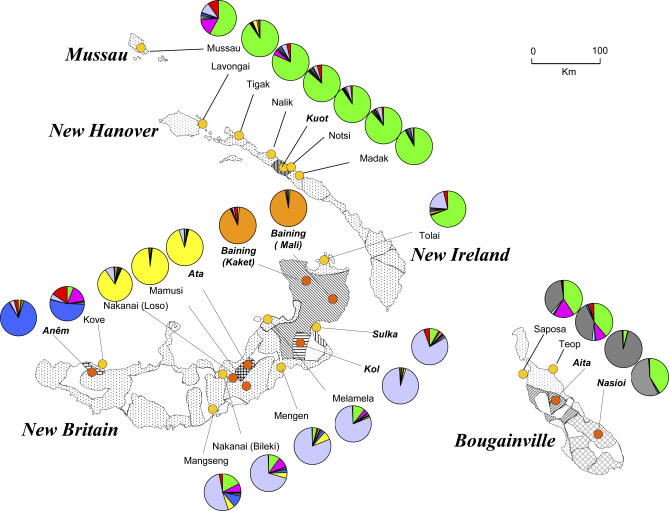
The Geographic Patterning of STRUCTURE Results Distribution of cluster assignment percentages (in pie-charts) among Northern Island Melanesian populations for *K=*10. Oceanic-speaking regions are stippled; the different Papuan-speaking regions have stripes or grid marks. Papuan-speaking group names are in bold italics. Inland group locations are dark orange dots; shore group locations are light orange dots. Baining (Mali) and Baining (Kaket) are two dialects; elsewhere, the two Kaket-speaking locales are identified (Rangulit and Malasait), as is Marabu (Mali-speakers).

We performed STRUCTURE analyses on a combined East Asia–Pacific dataset to explore in detail the relationships among Melanesians, Polynesians, Taiwan Aborigines, and East Asians, and to clarify the role of intermixture there. The samples included in this analysis were our Pacific set of 40 groups, and from the HGDP-CEPH panel, the “Papuans,” (identified here as “Highlands”), the East Asians, and French (the French were included to identify European admixture). The STRUCTURE results are shown in Figure 6, and the details on their reproducibility in Table S5. At *K* = 2 and *K* = 3, the Asia-Pacific clusterings mirrored the first five runs of the global comparison. Bougainville formed a cluster contrasting with central New Britain at *K =* 3; the New Guinea groups separate at *K* = 4; and a central New Britain cluster splits at *K =* 5. Then, at *K =* 6, a Polynesian cluster appeared, centered on the Mãori, with high ancestral proportions for the Samoan and Micronesian samples as well as the Taiwanese Aborigines. The former “East Asian” ancestral proportion in Melanesian populations converted almost entirely to “Polynesian” in this run. At *K* = 7, 8, and 9, more Melanesian clusters formed in New Britain and New Ireland. All but one of the Melanesian cluster foci are Papuan-speaking groups, primarily located in the interiors of the large islands (see Figures 7 and 8). The Mamusi, who are Oceanic-speaking neighbors of the Ata, are the exception. There is reason to suspect the Mamusi were originally a Papuan-speaking group (perhaps Ata speakers) who adopted an Oceanic language [34]. At *K* = 10, the “Europeans” were finally identified as a separate cluster. As shown in Table S5, runs at *K* = 11 and above became unstable and not reproducible.

The approximate percentage of “European” admixture is best seen in Figure 7, which gives average ancestral proportions by population. In the Mãori, the “European” ancestry was ∼12%, and for Samoans it was ∼5%. The Samoan and Micronesian results also suggested minor ties with East Asians and also Melanesians, specifically the “New Ireland” cluster (a number of Lapita sites have been found in the vicinity of New Ireland [3]). The Micronesians had low levels of inferred ancestry shared with populations in New Guinea, which is not far from Belau, where most of the Micronesian samples are from. This relationship is echoed in mtDNA results as well [35]. The typical ancestral proportions by population for a majority rule run are given in Table S6. As seen in Table S5, 15 out of 20 STRUCTURE runs on our Pacific dataset at *K* = 10 produced essentially the same group ancestry proportions as shown in Figures 6 and 7, with individual similarity coefficients ranging from 0.90 to 0.96, so these results are quite reproducible.

As in the global comparison, an “East Asian/Polynesian” estimated ancestry proportion for a number of Melanesian populations only occurred at frequencies of >5% in certain Oceanic-speaking (Austronesian) groups, and it is hereafter referred to as the “Austronesian” genetic signature. In Figure 7, the purple arrows point to those Oceanic-speaking groups in our Melanesian sample set that have this clear “Austronesian” signature. The probabilities were highest in the Kove and Saposa (just below 20%), followed by the Mussau at 15%, with the Teop, Mangseng, Nakanai (Bileki), Melamela, and Tigak having lower “Austronesian” signatures. In these Oceanic-speaking populations, the “Austronesian” ancestral assignment proportions never ranked higher than third, indicating their comparatively intermixed, and predominantly Papuan, genetic nature.

As a check on these results, particularly to verify the relationships of the Polynesians and Micronesians within our dataset, we performed a separate “supervised” STRUCTURE analysis [28,36], where the individual Mãori, Samoan, and Micronesian genotypes were distributed across eight representative populations (Taiwan Aborigines, East Asians, Europeans, and the Near Oceanic New Guinea, Ata, Baining, Kuot, and Aita). The results, shown in Figure S3A, underline the primary affinity of the Mãori, Samoans, and Micronesians to Taiwan Aborigines and secondarily to East Asians, with lesser suggestions of links to Europeans and New Ireland/New Britain (there is no suggestion of any Bougainville or Baining tie). In a second “supervised” STRUCTURE analysis where a ninth population was specified but not associated with a particular group a priori, the Polynesians/Micronesians constituted the largest proportion of this cluster (Figure S3B). Of the three populations in question, the Mãori had the smallest signal of external relationship, consistent with their extensive genetic drift, and the Micronesian group has the largest signal (to Taiwan, East Asia, New Guinea, and New Ireland/New Britain).

Figure 8 shows the distribution within Northern Island Melanesian populations of the STRUCTURE clustering probabilities for *K* = 10 in pie-chart form (some populations from the same language groups with very similar probability profiles were merged). Neighboring groups tended to share similar profiles. New Britain, the largest and most rugged island, had the greatest internal differentiation, with five different assigned clusters at >50% probabilities in different populations. Bougainville groups had two common cluster assignments, while there was only one common cluster in New Ireland.

**Figure 9:**
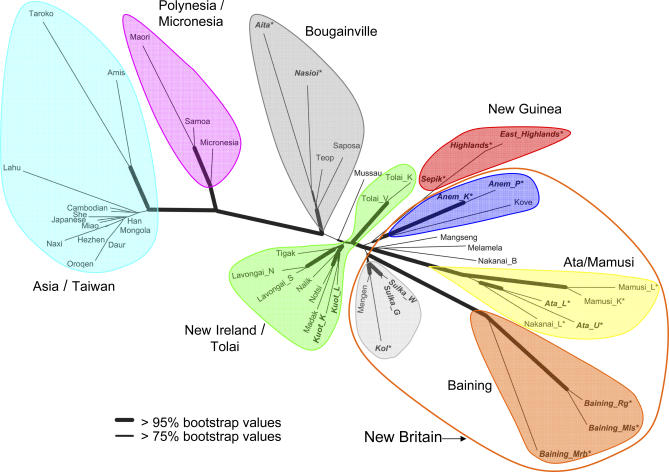
Pacific Population Tree Neighbor-joining *F*_ST_-based tree for 687 microsatellites from the Pacific, East Asia, and French populations, with the range of bootstrap values indicated by branch thicknesses. Colors are the same as in the STRUCTURE analysis at *K* = 10. New Britain populations are circled. Papuan-speaking groups are in bold italics; inland groups in Melanesia have asterisks. Abbreviated names are spelled out in Table S1.

Figure 9 shows the unrooted neighbor-joining tree for the East Asia–Pacific populations from a pairwise *F*_ST_ coancestry distance matrix for 687 microsatellites (the pairwise *F*_ST_ values are in Table S7). Bootstrap values for the branches, generated with the PHYLIP program from population allele frequencies for 100 different trees, are indicated by branch thicknesses. As shown, most of the trunk elements had high bootstrap values, as did a number of branches within Northern Island Melanesian groups. By contrast, the mainland East Asian group relationships were considerably more ambiguous, their branches were shorter, and only the Taiwan Aborigines had a strong internal branch. The tree branching again closely reflected the clustering in STRUCTURE, indicated by the corresponding colors from *K* = 10. The populations with the longest branches were those with the largest ancestral proportions assigned to single STRUCTURE clusters, and had the lowest heterozygosities. These populations tend to be Papuan-speaking groups in island interiors. The STRUCTURE analysis specifies the role and nature of admixture in a way that a population-based tree cannot.

The AMOVA, STRUCTURE, and population tree analyses were all driven by large distinctions in allele frequencies, rather than by the presence of private alleles in one population or another, since these generally occur in very low frequencies. In the first publication on the global HGDP-CEPH set of 377 microsatellites, Rosenberg et al. quantified continental relationships independent of the STRUCTURE analysis by showing the number of alleles that were only present in one continent, shared by two, by three, etc. [24]. The pattern of specific allele sharing was taken to indicate greater African heterogeneity, and that allele sharing was least for the Americas and for the two “Oceanic” groups.

With our enlarged dataset and microsatellite coverage, we also compared patterns of private alleles and allele sharing between regions (Table 3). We recovered 271 Melanesian-specific alleles, which in raw numbers actually exceeded those for Africa. Correcting for sample sizes, the rate of Melanesian-specific alleles was at the high end of the range for the major regions except for Africa. The number of alleles missing from only one continent, also given in Table 3, shows the dramatic effect of genetic drift on the American populations. The number of shared alleles between pairs of regions is shown in Table 4, with the correction for sample sizes in Table 5. All non-African regions including Melanesia shared the most alleles with Africa, indicating they were primarily subsets of African diversity. Melanesia shared more alleles with East Asia than with any other non-African region, but they cannot simply be viewed as an extension or subset of East Asian diversity. When Papuan and Oceanic speaking groups in Melanesia were analyzed separately, the Papuan-speaking groups showed greater isolation, as they shared fewer alleles with all other regions than did Oceanic speaking groups (unpublished data).

**Table 3:**
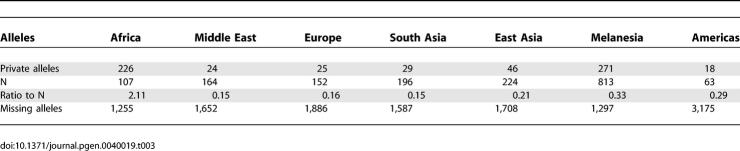
Private and Missing Alleles by Continent

**Table 4:**
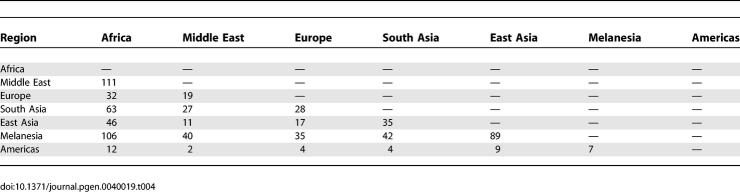
Bi-Continental Allele Sharing

**Table 5:**
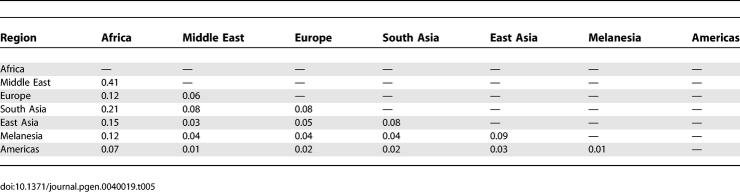
Bi-Continental Allele Sharing, Corrected by Combined Sample Sizes

## Discussion

### Language and Genetic Correspondences

Our study suggests that in the Pacific, and specifically in Near Oceania, there is only a modest association between language and genetic affiliation. Oceanic languages were introduced and dispersed around the islands within the last 3,300 years, but there was apparently only a small infusion of accompanying “Austronesian” ancestry that has survived. Approximately one-half of the Oceanic-speaking groups in Melanesia had an identifiable “Austronesian” genetic signature (see Figure 7 and Table S8). In each case where there was such an “Austronesian” signature, at least two other cluster assignments had probabilities higher than the “Austronesian” one (see, in Figure 6, the Saposa and Teop of Bougainville; the Mussau and Tigak in New Ireland Province; and the Kove, Mangseng, Melamela, and Nakanai Bileki of New Britain). On the other hand, the Oceanic-speaking groups without the “Austronesian” signature were often genetically indistinguishable from their immediate Papuan-speaking neighbors (in New Britain, the Mamusi have no Austronesian signature, but they and the Nakanai Loso cluster closely with their Papuan-speaking Ata neighbors; the Nalik, Notsi, and Madak of New Ireland are genetically indistinguishable from their Papuan-speaking Kuot neighbors; the Tolai and Lavongai profiles suggest significant intermixture, but only between different Papuan-speaking groups). The result suggests that Oceanic languages were adopted by many formerly Papuan-speaking groups, while at the same time there was little genetic influence or marital exchange. At least in Near Oceania, rates of language borrowing and language adoption have been faster and more pervasive than rates of genetic admixture.

### Melanesians in the Global Context

**Figure 10:**
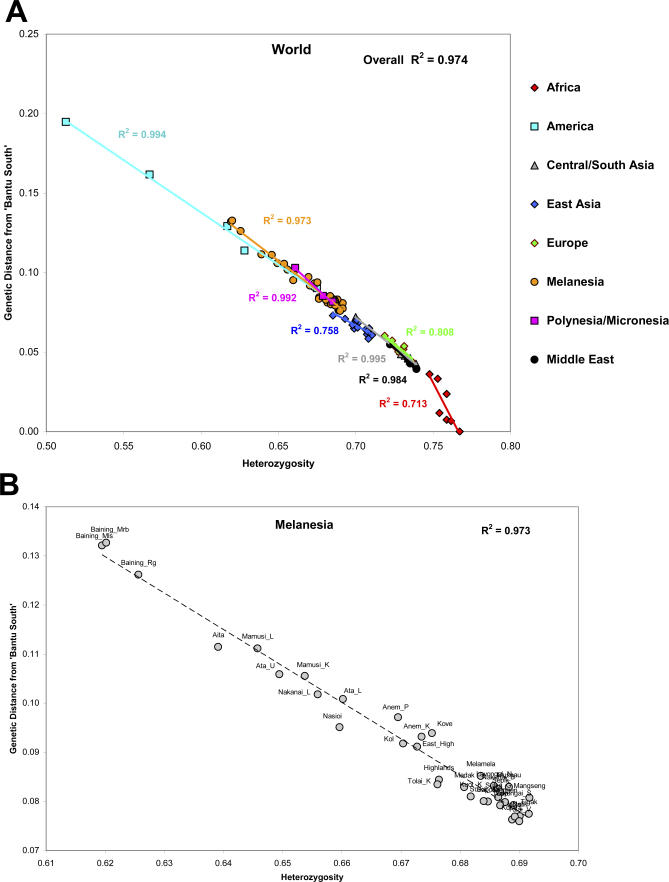
The Correlation between Genetic Distances and Heterozygosity The genetic distances used were the set of pairwise *F*_ST_s involving Bantu South (the population with the highest heterozygosity), highlighted in Table S4. (A) The combined global dataset. (B) Details for Melanesia.

However it is measured, genetic variation is reduced within Melanesian populations (Figure 2), while the genetic divergences among them are very large (refer to Figures 6, 8, and 9 and to Tables 1–5). The size of the differences among the populations would appear to equal or surpass those among populations across East Asia, Europe, or even Africa. However, the large Melanesian population distinctions are a direct consequence of their very low levels of internal variation or heterozygosity. These low levels will directly inflate both the proportion of among group variation in AMOVA and also pairwise *F*_ST_ genetic distances (for a full discussion of this point, see especially [27] and also [ 26,37]). As population heterozygosities decrease, pairwise *F*_ST_s should increase because of this intrinsic mathematical relationship. This is illustrated by our global and Near Oceania datasets (Figure 10A and 10B). Those pairwise *F*_ST_s involving the Bantu South population (which has a heterozygosity approaching 1.0) are plotted against the heterozygosities of each population, and the resulting correlations approach 1.0.

Our Structure and tree analyses of the combined microsatellite datasets indicate that Melanesians are quite far removed from Africans, in spite of their superficial similarities in hair form and skin pigmentation [38]. In the initial analysis of the HGDP-CEPH dataset, the placement of the two Melanesian (“Oceanic”) groups was different. There, they split from Eurasia before Asians and Native Americans [39]. This also differed from the result of a genome-wide SNP study [40] on a very small world-wide dataset. The extreme positioning of Melanesians in our tree was not due to our over sampling. Rather, our extensive coverage of Melanesian variation has enabled a clearer resolution of their relationships with populations outside the region.

### The Causes of Melanesian (Near Oceanic) Diversity

The pattern of Near Oceanic diversity has been made clear. The AMOVA analysis of the microsatellites showed that the larger and more rugged the island, the greater the differentiation among populations. The most divergent populations were in large island interiors while these same populations were internally the most homogeneous (as measured by reduced values of *θ̂* and expected heterozygosity—Table S1). Genetic variation from one large Near Oceanic island to the next was also significant. While our coverage of microsatellite variation elsewhere in the Pacific was admittedly spotty, our data as well as other smaller scale microsatellite analyses [21,41] suggest that, excluding the large islands of Near Oceania, there is a gradual decline in variation as one moves from Asia eastward, and variation among populations in the Pacific otherwise is not nearly as great as that in the large islands of Near Oceania. As noted, New Guinea does not appear to have as much microsatellite/indel diversity among groups as New Britain. Our sample coverage and definition was less rigorous there, and we expect equivalent coverage in New Guinea would equal or surpass the divergence of our New Britain series.

The biogeographic pattern of population divergences in Near Oceania is most likely attributable to the restricted marital migration distances that have been documented most clearly for inland Bougainville groups [42], as well as for some New Guinea highlands populations [43]. Few people in small inland communities traditionally married and established households more than 1–2 kilometers from their birthplaces, while marital migration distances tended to be longer among shoreline communities. Nettle has argued that in ecologically rich tropical regions such as Near Oceania, small populations easily became self-sufficient, which in turn encouraged isolation and discouraged exchange [44,45], causing the development of extreme diversity among populations in both language and genetics. We suggest this was the underlying cause of the short marital migration distances among inland groups in Near Oceania, which in turn was responsible for the low population heterozygosities and resulting large genetic distinctions among groups [42].

Because they arrived first and came to occupy large island interiors, the Papuan-speaking groups are considerably more diverse than Oceanic-speaking groups, which tend, in large islands, to be arranged along the shorelines. The prehistoric record suggests there was a gradual reduction after initial settlement in the size of foraging zones of formerly mobile groups, associated with the filling up of the landscape [3, p. 16]. In many ways, these patterns and dynamics parallel the biogeography of birds and ants in the same region, where dispersal abilities of different species have dictated their patterns of diversity, and dispersal tendencies have, in many cases, contracted in island interiors over time [46,47].

Some known population relationships suggested the considerable age of the clusters identified by our STRUCTURE analysis. The Tolai of East New Britain, with an assignment profile similar to New Ireland groups, are known to have migrated from southern New Ireland over 1,200 years ago [42]. A major volcanic eruption in western New Britain 3,000 years ago isolated that section of the island, and the Anêm, along with the recently arrived and intermixed Kove, form a separate cluster. Although the two Baining groups of east New Britain formed a cluster of their own, it has been suggested from the mtDNA, Y, and X chromosome analyses that they have been separated by thousands of years [48] (see their long branch lengths in Figure 9). Also, the clustering of the Polynesians, Taiwan Aboriginals, and East Asians reflects ties older than 3,300 years. In the Pacific, the change in genetic clustering apparently has evolved over thousands of years, and in many cases tens of thousands. This is likely a function of small effective population sizes and the high degree of isolation/drift over these immense time periods.

### The Origins of Polynesians—“The Genetic Trail”

There were indications from the mtDNA, NRY, and certain autosomal microsatellites that in Remote Oceania, where islands are generally smaller in size, genetic variation among human groups is comparatively reduced, which is a contrast to Near Oceania [17,19–21,49]. At some point, prehistoric Oceanic mariners apparently became so accomplished that the inter-island water crossings in the central Pacific were often no more of an impediment to travel than the (already occupied) rugged terrain of the larger island interiors in the western Pacific. In many areas, the ocean was transformed from a formidable barrier into a highway [50,51].

However, exactly where the (relatively homogeneous) Polynesians came from has remained controversial, and the number of proposed explanatory models for their origin form a continuum [49,52]. At one extreme is the “Entangled Bank” [53], which is essentially a null hypothesis for detecting clear signals of specific Polynesian ancestry anywhere to the west. It suggests that, although there certainly must have been a series of introductions and influences from Asia into the Pacific over the millennia, no decipherable signal has survived that can be identified as specifically ancestral to Polynesians, because of the complexities of human interactions from the outset [54]. Proponents argue that tree-like representations of population (or linguistic) relationships cannot be expected to develop regularly and are likely to be entirely inappropriate representations of population relationships in many, if not all, instances, since they so often ignore interactions between neighboring groups.

Models at the other end of the continuum assume contemporary genetic (as well as cultural) similarities can carry a clear signal of past population relationships. Primary among these is “The Express Train to Polynesia” model [55]. It proposes a rapid movement of the ancestors of the Polynesians from the vicinity of Taiwan to the Central Pacific, without extensive contact with indigenous Near Oceanic populations along the way.

With regard to human genetics, the published mtDNA evidence has generally been interpreted as supporting the “Express Train.” This is because a younger mtDNA haplotype (B4a1a1) is assumed to have been closely linked to the development and expansion of Polynesian populations. At present, the state of the evidence for this association is as follows: a) the precursor haplotype to B4a1a1 has been identified in Taiwan aboriginal populations [56]; b) the final development of B4a1a1 with the key mutation at nucleotide site 14022 seems to have occurred in eastern Indonesia or Near Oceania [17]; c) its frequency varies widely over Near and Remote Oceania before becoming ubiquitous in Central and Eastern Polynesian populations; d) in Near Oceania, it is common along many Oceanic-speaking coastal groups, as well as a number of Papuan-speaking groups, especially in New Ireland and Bougainville [17]; and e) its expansion dates are relatively recent, although old enough to suggest to some observers that it cannot be easily tied to the Polynesian expansion [17,56].

The “Slow Boat to Polynesia” model which is supported by NRY variant distributions, also assumes current genetic patterns in Oceania directly reflect prehistoric migrations and interactions. These NRY haplogroup distributions have been taken to suggest a very minor “Asian” contribution to current Polynesian populations, suggesting instead that Polynesians derived primarily from Melanesian (Near Oceanic) populations [19,57,58]. “Melanesian” NRY haplogroups were found to be very common in some Polynesian populations, while “Asian” NRY haplogroups were scarce in Melanesian populations [20,58], and low in their frequencies in the Central Pacific. However, recent studies have shown that the “Asian” NRY haplogroups are not as rare in Polynesia as initially thought, and are quite variable in frequency ([19], Table S2).

Because of their comprehensive nature, we believe the results of our autosomal microsatellite survey present a resolution to this issue with regard to human genetic relationships. The fact that the STRUCTURE cluster containing Micronesians, Samoans, and Maoris has a detectable signature only in Oceanic-speaking Melanesians and Taiwan Aborigines supports the position that an expansion of peoples from the general vicinity of Taiwan is primarily responsible for the ancestry of Remote Oceania, and that these people left a small but still identifiable signature in (some Oceanic-speaking) populations of Near Oceania. Scenarios for different male and female dispersals have been proposed to reconcile the divergent mtDNA and NRY patterns in Oceania [35,59], but the autosomal microsatellite results should now serve as the primary reference.

Although the Polynesians in our analysis were similar to Taiwan Aborigines and East Asians, they might be even closer to other populations not covered in our study, from Indonesia, the Philippines, or Southeast Asia. While there is a substantial body of evidence that indicates Taiwan is the primary point of Austronesian dispersal [60,61], there are now also suggestions of the importance of (Island) Southeast Asia as well [62,63]. The ties of particular Near Oceanic populations to those regions also remain poorly understood, but should be resolved with additional sampling from these regions and similar analyses.

### Conclusion

To revisit the questions posed at the beginning, we can provide answers as follows.

1) *To whom are these Melanesian populations most closely related outside the Pacific?* Outside the Pacific, East Asian populations are apparently the closest (but still very distant) relatives of Melanesians. Africans and Europeans are the most distant.

2) *How does the genetic diversity of Near Oceanic populations compare with groups in other regions?* The within-group diversity in Melanesian populations is consistently very low, which acts to exaggerate the considerable among-group distinctions there. This great diversity in such a small region makes comparisons of human population structure from continent to continent problematic.

3) *Is there a clear organization of the variation among Melanesian groups?* The diversity among groups is primarily organized by island size and topographic complexity, with the inland Papuan-speaking groups the most isolated and differentiated. Shore-dwelling Oceanic-speaking groups are more intermixed (dispersal along the shorelines was easier).

4) *Is there an identifiable genetic signature of Taiwanese/Southeast Asian or Polynesian influence in Near Oceanic populations, especially in the Bismarcks, where the Lapita Cultural Complex developed?* There is a weak “Austronesian” genetic signature in only a portion of Oceanic-speaking populations in Melanesia, and none at all in Papuan-speaking groups (contradicting the results of mtDNA, but in accord with the NRY results).

5) *Are Polynesians more closely related to Asian/Taiwanese populations or to Melanesians?* Polynesians are closely related to Asian/Taiwanese Aboriginal populations, while they are very weakly associated with any Melanesian groups (the closest association there appears to be with New Ireland populations). This is in accord with mtDNA interpretations, but differs from the usual interpretation of the NRY results. The sailing capabilities of the ancestors of the Polynesians transformed the nature of their Diaspora and kept them relatively homogeneous.

## Methods

### Sampled individuals.

Our Asia–Pacific sample set came from a variety of sources. The objective was to include between 15 and 25 unrelated individuals (minimally excluding reported first-degree relatives) from locales where individuals and their parents had all lived. These criteria were achieved in most instances. All of the samples except the cell lines were Whole Genome Amplified (Qiagen RepliG). Details are given below.

1. Samples from Northern Island Melanesia were collected in three field seasons (1998, 2000, and 2003) in collaboration with the Institute for Medical Research of Papua New Guinea. Besides a 10 ml blood sample, a simple genealogy and residency questionnaire was taken, including in most instances parent and grandparent names, residences, and native languages. All individuals gave their informed consent for participation, and the study was approved by the Institutional Review Boards of Papua New Guinea, Temple, Michigan, Yale, and Binghamton Universities. Among over 1,500 samples collected, 995 were chosen for submission to the Marshfield Clinic for microsatellite and indel analysis. As many Papuan-speaking groups as possible were included, along with neighboring Oceanic-speaking groups, focusing on New Britain, New Ireland, New Hanover, Mussau, and Bougainville. We included multiple locales in larger language groups where feasible; and picked samples from individuals whose family's residence histories suggested close identification with the sampling locale. People of mixed parentage (especially with one grandparent from a different language group or island) could not always be excluded if the minimum required sample size was to be achieved. A number of individuals who were born on the New Guinea mainland but had settled in Northern Island Melanesia were taken to constitute one additional sample—the “Sepik” —so that this sample is a conglomerate. DNA was extracted as previously described [17].

2. DNA was obtained from the Kidd lab collection of cell lines for: a) the Eastern Highlands of Papua New Guinea, primarily from the Gimi, which were collected in collaboration with the Papua New Guinea Institute of Medical Research, and also from Goroka Town; b) Micronesians, primarily from Belau, who drew each other's blood samples during their training in the Pacific Basin Medical Officer Training Program; and c) Samoans, who were in a combined collection from the Pacific Basin Medical Officer Training Program and from American Samoa. All individuals gave their informed consent for participation.

3. New Zealand DNA samples were collected from indigenous Mãori individuals residing in the North Island. Individuals were unrelated by first degree, had two Mãori parents by self-report, and belonged to one segment of the wider Mãori population. Ethical clearance was granted by the NZ National Ethics Committee. DNA was extracted from blood using Qiagen kits.

4. Taiwan Aboriginal samples comprise the Northeastern Taroko tribe from Hsiulin, part of the Atayal language group, and the Amis tribe living on the east coast of Taiwan and speaking Amis. All individuals were unrelated and had both parents belonging to the same tribe. Each individual gave informed consent to participation in population genetics studies and the project was approved by the Ethics Committee of the Hospital and the Department of Health of Taiwan. Blood samples were collected in acid citrate dextrose tubes. Genomic DNA was extracted from 500 μl of buffy coat using the QIAmp DNA kit (QIAml blood kit, Qiagen) by Loo Jun-Hun at the Transfusion Medicine and Molecular Anthropology Laboratory, Mackay Memorial Hospital, Taipei.

### Markers.

Each individual was originally genotyped for 751 microsatellite and 481 insertion/deletion autosomal polymorphisms. The microsatellites were drawn from Marshfield Screening Sets #16 and #54, and the indel markers were drawn from Marshfield Screening Set #101.

### Combined dataset including the HGDP-CEPH Human Genome Diversity Cell Line Panel.

890 markers typed in our Pacific series (203 indels and 687 microsatellites) had been typed in the HGDP-CEPH Human Genome Diversity Cell Line as described in [23], although for some microsatellites, a change in primer length or position occurred between the HGDP-CEPH genotyping (2004) and our own (2006), or a change in allele calling occurred. Where the primer changed, allele sizes from one of the two data sets were adjusted (Table S9). The changes were done by comparing the same set of individuals (called “Nasioi” in our dataset, and “Melanesians from Bougainville” in the HGDP-CEPH dataset) duplicated in both studies. Two loci for which the allele size shift was ambiguous—GATA11C08 and GGAA10C09—were excluded. Of the 687 microsatellites remaining for the combined analysis with the HGDP-CEPH panel, 166 had primer changes between the datasets. All analyses utilized the 687 microsatellites, and in addition the 203 indels were used in the STRUCTURE analyses. The set of 957 individuals used here from the HGDP-CEPH panel is the “H971” subset of the original panel [64], without first-degree relatives, and with the Melanesian (Nasioi) removed, since these individuals were also present in our samples (one individual, number 857, was inadvertently deleted early in this analysis). Small African populations with single or two individuals were grouped into Bantu South (Herero, Ovambo, Pedi, Sotho, Tswana, and Zulu)

### Population genetic analysis.

The expected heterozygosity and average number of alleles per locus were computed on the microsatellites with the GDA software [65], using the sample-size corrected estimator, as in [66]. *F*_ST_ was estimated on the microsatellites as in Equation 5.3 from [67] , using GDA, with 95% confidence intervals based on 1,000 bootstraps across loci. Indels were excluded from all analyses except STRUCTURE.

Cluster analysis of genotypes utilized the Structure versions 2.1 and 2.2 software package [28,36]. Results using Structure 2.1 and 2.2 were essentially identical. STRUCTURE was run with a Markov Chain Monte Carlo (MCMC) burnin of 20,000 steps, followed by an MCMC chain of 10,000 steps for clustering inference. Ten runs were performed at each *K* in most cases, except as noted in Table S3 (for *K* = 7) and Table S5 (for *K* = 10). When multiple runs at the same values of *K* produced discrepant results, we relied on majority rule (i.e., modal topography in cluster assignment) to pick the optimal result. For the combined global analysis, we terminated the STRUCTURE runs at *K* = 6, as explained in the Results, and for the Pacific set we terminated the analysis when it became unstable at higher values of *K* (i.e., when multiple solutions appeared). Details are provided in the Tables S3 and S5.

Individual similarity coefficients for pairs of runs were calculated as in [24] and Methods.

The neighbor-joining trees for Figures 5 and 9 were based on the *F*_ST_ distance matrices obtained with GDA. The bootstrap values for the Asia–Pacific dataset (Figure 9) were obtained based on allele frequencies using PHYLIP [68]. The neighbor-joining trees in Figure S3 were calculated using MSA [69] and drawn with Phylip.

Great circle geographic distances were calculated with the Haversine method as described in [26].

The results of the STRUCTURE runs were graphed with the software DISTRUCT [70].

## Supporting Information

Figure S1 The Divisions of Austronesian Languages The relationships are shown for, among others, the Taiwanese languages, Malayo-Polynesian, Proto Oceanic, Micronesian, Polynesian, and the Oceanic languages of Island Melanesia and New Guinea. After Blust [10,71,72] and Pawley (personal communication). Some relevant island specifications: Southeast Solomonic (Malaita, Makira, Guadalcanal); Te Motu (Santa Cruz, Reefs, Vanikolo, Utupua); Meso-Melanesian (NE New Britain plus Bali-Witu, New Ireland, Bougainville, Western Solomons).
(762 KB TIFF)

Figure S2 Neighbor-Joining Trees for the Combined CEPH-HDGP and Pacific Datasets, Using Various Pairwise Distance Statistics (A) Cavalli-Sforza and Edwards' Chord Distance [33].
(B) Goldstein's (δ*μ*)^2^ [31].
(C) Nei's chord distance [30].
(D) Proportion of shared alleles (PSA) [73].
(9.3 MB TIF)

Figure S3 “Supervised” STRUCTURE Analysis, with the Mãori, Samoans, and Micronesians,and Eight Specified Representative Populations (Europeans [French], East Asians, Taiwan Aborigines, New Guinea, New Britain [Ata and Baining], New Ireland [Kuot], and Bougainville [Aita]) The Mãori, Samoan, and Micronesian individual profiles are compared with eight specified representative populations (Europeans [French], East Asians, Taiwan Aborigines, New Guinea, New Britain [Ata and Baining], New Ireland [Kuot], and Bougainville [Aita]).
(A) The distribution of the Mãori, Samoans, and Micronesians across the eight specified groups, at *K* = 8 (by individual and population proportions).
(B) The same samples and restrictions with an extra, unspecified, cluster (*K* = 9).
(4.3 MB TIF)

Table S1 Sample Descriptions Language assignments, sample sizes, expected heterozygosity (*H_e_*), estimated *θ* (*θ*), and mean alleles per locus.
(34 KB XLS)

Table S2 AMOVA Results for Melanesian Islands on 687 Microsatellites (16 KB XLS)

Table S3 Reproducibility of STRUCTURE Runs on the Combined Datasets (15 KB XLS)

Table S4 Matrix of Pairwise *F*_ST_ “Coancestry” Distances (or Reynolds' D) for the Combined HGDP-CEPH and Pacific Datasets (92 KB XLS)

Table S5 Reproducibility of STRUCTURE Runs on the Asia–Pacific Dataset (15 KB XLS)

Table S6 Cluster Assignment Probabilities (*K* = 10) of Pacific Populations, plus HGDP-CEPH East Asian and French Samples (30 KB XLS)

Table S7 Asia–Pacific Pairwise *F*_ST_ Coancestry Distance Matrix (45 KB XLS)

Table S8 Austronesian Coancestry Proportions across 15 Most Consistent Runs (Similarity Coefficients > 0.90) (39 KB XLS)

Table S9 Changes to Allele Sizes (29 KB XLS)

## References

[pgen-0040019-b001] Friedlaender JS (2007). Genes, language, and culture history in the Southwest Pacific.

[pgen-0040019-b002] Green RC, Pawley A (1991). Near and remote Oceania—Disestablishing “Melanesia” in culture history. Man and a half: Essays in Pacific anthropology and ethnobiology in honour of Ralph Bulmer.

[pgen-0040019-b003] Summerhayes GR, Friedlaender JS (2007). Island Melanesian Pasts—A view from archaeology. Genes, language, and culture history in the Southwest Pacific.

[pgen-0040019-b004] Wickler S, Spriggs M (1988). Pleistocene human occupation of the Solomon Islands, Melanesia. Antiquity.

[pgen-0040019-b005] Leavesley M, Chappell J (2004). Buang Merabak: additional early radiocarbon evidence of the colonisation of the Bismarck Archipelago, Papua New Guinea. Antiquity Project Gallery.

[pgen-0040019-b006] Summerhayes GR, Chiu S, Sand C (2007). The rise and transformations of Lapita in the Bismarck Archipelago. From Southeast Asia to the Pacific: Archaeological perspectives on the Austronesian expansion and the Lapita cultural complex.

[pgen-0040019-b007] Swadling P, Hide R, Pawley A, Attenborough R, Golson J, Hide R (2005). Changing landscape and social interaction: looking at agricultural history from a Sepik–Ramu perspective. Papuan pasts: Cultural, linguistic and biological histories of Papuan-speaking peoples.

[pgen-0040019-b008] Anderson A, Clark GR, Anderson AJ, Vunidilo T (2001). Mobility models of Lapita migration. The archaeology of Lapita dispersal in Oceania papers from the Fourth Lapita Conference.

[pgen-0040019-b009] Kirch PV (1997). The Lapita people: Ancestors of the Oceanic world.

[pgen-0040019-b010] Blust R (1995). The prehistory of the Austronesian speaking peoples: a view from language. J World Prehistory.

[pgen-0040019-b011] Ross M, Pawley A, Attenborough R, Golson J, Hide R (2005). Pronouns as a preliminary diagnostic for grouping Papuan languages. Papuan Pasts: Investigations into the cultural, linguistic and biological history of the Papuan speaking peoples.

[pgen-0040019-b012] Pawley A, Pawley A, Attenborough R, Golson J, Hide R (2005). The chequered career of the Trans New Guinea Phylum: Recent historical research and its implications. Papuan pasts: Investigations into the cultural, linguistic and biological history of the Papuan speaking peoples.

[pgen-0040019-b013] Dunn M, Terrill A, Reesink G (2002). The East Papuan languages: A preliminary typological appraisal. Oceanic Linguistics.

[pgen-0040019-b014] Lindström E, Terrill A, Reesink G, Dunn M, Friedlaender JS (2007). The languages of Island Melanesia. Genes, language, and culture history in the Southwest Pacific.

[pgen-0040019-b015] Pawley A, Friedlaender JS (2007). Recent research in historical relationships of the Papuan languages: or, what does linguistics say about the prehistory of Melanesia?. Genes, language, and culture history in the Southwest Pacific.

[pgen-0040019-b016] Pierson MJ, Martinez-Arias R, Holland BR, Gemmell NJ, Hurles ME (2006). Deciphering past human population movements in Oceania: Provably optimal trees of 127 mtDNA genomes. Mol Biol Evol.

[pgen-0040019-b017] Friedlaender JS, Friedlaender FR, Hodgson JA, Stoltz M, Koki G (2007). Melanesian mtDNA Complexity. PLoS ONE.

[pgen-0040019-b018] Friedlaender JS, Friedlaender FR, Hodgson J, McGrath S, Stoltz M, Friedlaender JS (2007). Mitochondrial DNA Variation in Northern Island Melanesia. Genes, language, and culture change in the Southwest Pacific.

[pgen-0040019-b019] Kayser M, Brauer S, Cordaux R, Casto A, Lao O (2006). Melanesian and Asian origins of Polynesians: mtDNA and Y chromosome gradients across the Pacific. Mol Biol Evol.

[pgen-0040019-b020] Scheinfeldt L, Friedlaender F, Friedlaender J, Latham K, Koki G (2006). Unexpected NRY chromosome variation in Northern Island Melanesia. Mol Biol Evol.

[pgen-0040019-b021] Lum JK, Jorde LB, Schiefenhovel W (2002). Affinities among Melanesians, Micronesians, and Polynesians: a neutral biparental genetic perspective. Hum Biol.

[pgen-0040019-b022] Cann HM, de Toma C, Cazes L, Legrand MF, Morel V (2002). A human genome diversity cell line panel. Science.

[pgen-0040019-b023] Rosenberg NA, Mahajan S, Ramachandran S, Zhao C, Pritchard JK (2005). Clines, clusters, and the effect of study design on the inference of human population structure. PLoS Genet.

[pgen-0040019-b024] Rosenberg N, Pritchard J, Weber J, Cann H, Kidd K (2002). Genetic structure of human populations. Science.

[pgen-0040019-b025] Ohta T, Kimura M (1973). A model of mutation appropriate to estimate the number of electrophoretically detectable alleles in a finite population. Genetic Research.

[pgen-0040019-b026] Ramachandran S, Deshpande O, Roseman CC, Rosenberg NA, Feldman MW (2005). Support from the relationship of genetic and geographic distance in human populations for a serial founder effect originating in Africa. Proc Natl Acad Sci U S A.

[pgen-0040019-b027] Long JC, Kittles RA (2003). Human genetic diversity and the non-existence of biological races. Human Biology.

[pgen-0040019-b028] Pritchard JK, Stephens M, Donnelly P (2000). Inference of population structure using multilocus genotype data. Genetics.

[pgen-0040019-b029] Reynolds J, Weir BS, Cockerham CC (1983). Estimation of the coancestry coefficient: Basis for a short-term genetic distance. Genetics.

[pgen-0040019-b030] Nei M, Tajima F, Tateno Y (1983). Accuracy of estimated phylogenetic trees from molecular data. II. Gene frequency data. J Mol Evol.

[pgen-0040019-b031] Goldstein D, Ruiz Linares A, Cavalli-Sforza LL, Feldman MW (1995). Genetic absolute dating based on microsatellites and the origin of modern humans. Proc Natl Acad Sci U S A.

[pgen-0040019-b032] Mountain JL, Cavalli-Sforza LL (1997). Multilocus genotypes, a tree of individuals, and human evolutionary history. Am J Hum Genet.

[pgen-0040019-b033] Cavalli-Sforza LL, Edwards AW (1967). Phylogenetic analysis. Models and estimation procedures. Am J Hum Genet.

[pgen-0040019-b034] Reesink G (2007). Personal communication.

[pgen-0040019-b035] Lum JK, Cann RL (2000). mtDNA lineage analyses: origins and migrations of Micronesians and Polynesians. Am J Phys Anthropol.

[pgen-0040019-b036] Falush D, Stephens M, Pritchard JK (2003). Inference of population structure using multilocus genotype data: linked loci and correlated allele frequencies. Genetics.

[pgen-0040019-b037] Charlesworth B (1998). Measures of divergence between populations and the effect of forces that reduce variability. Mol Biol Evol.

[pgen-0040019-b038] Norton HL, Friedlaender JS, Merriwether DA, Koki G, Mgone CS (2006). Skin and hair pigmentation variation in Island Melanesia. Am J Phys Anthropol.

[pgen-0040019-b039] Zhivotovsky LA, Rosenberg NA, Feldman MW (2003). Features of evolution and expansion of modern humans, inferred from genomewide microsatellite markers. Am J Hum Genet.

[pgen-0040019-b040] Shriver MD, Mei R, Parra EJ, Sonpar V, Halder I (2005). Large-scale SNP analysis reveals clustered and continuous patterns of human genetic variation. Hum Genomics.

[pgen-0040019-b041] Lum JK, Friedlaender JS (2007). Contributions of population origins and gene flow to the diversity of neutral and malaria selected autosomal genetic loci of Pacific Island populations. Genes, language, and culture history in the Southwest Pacific.

[pgen-0040019-b042] Friedlaender JS, Friedlaender JS (2007). Introduction. Genes, language, and culture history in the Southwest Pacific.

[pgen-0040019-b043] Long JC, Naidu JM, Mohrenweiser HW, Gershowitz H, Johnson PL (1986). Genetic characterization of Gainj- and Kalam-speaking peoples of Papua New Guinea. Am J Phys Anthropol.

[pgen-0040019-b044] Nettle D, Harriss L (2003). Genetic and linguistic affinities between human populations in Eurasia and West Africa. Hum Biol.

[pgen-0040019-b045] Nettle D (1999). Linguistic diversity.

[pgen-0040019-b046] Mayr E, Diamond J (2001). The birds of Northern Melanesia: speciation, ecology, and biogeography.

[pgen-0040019-b047] Wilson EO (1961). The nature of the taxon cycle in the Melanesian ant fauna. Am Nat.

[pgen-0040019-b048] Wilder JA, Hammer MF, Friedlaender JS (2007). Extraordinary population structure among the Baining of New Britain. The history of genes, language, and culture in the Southwest Pacific: A synthesis.

[pgen-0040019-b049] Hurles ME, Matisoo-Smith E, Gray RD, Penny D (2003). Untangling Pacific settlement: On the edge of the knowable. Trends Ecol Evol.

[pgen-0040019-b050] Matisoo-Smith E, Friedlaender JS (2007). Animal translocations, genetic variation and the human settlement of the Pacific. Genes, language, and culture history in the Southwest Pacific.

[pgen-0040019-b051] Matisoo-Smith E, Roberts RM, Irwin GJ, Allen JS, Penny D (1998). Patterns of prehistoric human mobility in polynesia indicated by mtDNA from the Pacific rat. Proc Natl Acad Sci U S A.

[pgen-0040019-b052] Green RC, Sand C (2003). The Lapita horizon and traditions—Signature for one set of Oceanic migrations. Pacific archaeology: Assessments and anniversary of the first Lapita Excavation (July 1952), Koné, Nouméa, 2002.

[pgen-0040019-b053] Terrell J (1988). History as a family tree, history as an entangled bank: Constructing images and interpretations of prehistory in the South Pacific. Antiquity.

[pgen-0040019-b054] Terrell J, Kelly KM, Rainbird P (2001). “Foregone conclusions.” An analysis of the concepts of ‘Austronesians and ‘Papuans'. Curr Anthropology.

[pgen-0040019-b055] Diamond JM (1988). Express train to Polynesia. Nature.

[pgen-0040019-b056] Trejaut JA, Kivisild T, Loo JH, Lee CL, He CL (2005). Traces of archaic mitochondrial lineages persist in Austronesian-speaking Formosan populations. PLoS Biol.

[pgen-0040019-b057] Kayser M, Brauer S, Weiss G, Schiefenhovel W, Underhill PA (2001). Independent histories of human Y chromosomes from Melanesia and Australia. Am J Hum Gen.

[pgen-0040019-b058] Kayser M, Brauer S, Weiss G, Underhill PA, Roewer L (2001). Melanesian origin of Polynesian Y-chromosomes. Current Biology.

[pgen-0040019-b059] Hage P, Marck JC (2003). Matrilineality and the Melanesian origin of Polynesian Y chromosomes. Current Anthropology.

[pgen-0040019-b060] Gray RD, Jordan FM (2000). Language trees support the express-train sequence of Austronesian expansion. Nature.

[pgen-0040019-b061] Bellwood P, Fox JJ, Tryon D, Bellwood P, Fox JJ, Tryon D (1995). The Austronesians in history: Common origins and diverse transformations. The Austronesians: Historical and comparative perspectives.

[pgen-0040019-b062] Hill C, Soares P, Mormina M, Macaulay V, Clarke D (2007). A mitochondrial stratigraphy for island southeast Asia. Am J Hum Genet.

[pgen-0040019-b063] Larson G, Cucchi T, Fujita M, Matisoo-Smith E, Robins J (2007). Phylogeny and ancient DNA of Sus provides insights into neolithic expansion in Island Southeast Asia and Oceania. Proc Natl Acad Sci U S A.

[pgen-0040019-b064] Rosenberg NA (2006). Standardized subsets of the HGDP-CEPH Human Genome Diversity Cell Line Panel, accounting for atypical and duplicated samples and pairs of close relatives. Ann Hum Genet.

[pgen-0040019-b065] Lewis PO, Zaykin D (2001). GDA (Genetic Data Analysis): Computer program for the analysis of allelic data. Version 1.0 d16c ed.

[pgen-0040019-b066] Nei M (1987). Molecular evolutionary genetics.

[pgen-0040019-b067] Wier BS (1996). Genetic data analysis II: Methods for discrete population genetic data.

[pgen-0040019-b068] Felsenstein J (2005). PHYLIP (Phylogeny Inference Package). 3.66 ed.

[pgen-0040019-b069] Dieringer D, Schlötterer C (2003). Microsatellite analyser (MSA): a platform independent analysis tool for large microsatellite data sets. Molecular Ecology Notes.

[pgen-0040019-b070] Rosenberg N (2004). Distruct: a program for the graphical display of population structure. Molecular Ecology Notes.

[pgen-0040019-b071] Blust R, Jen-Kuei P, Tsang LC, Huang Y, Lo D-A, Tseng C-Y (1995). The position of the Formosan languages: method and theory in Austronesian comparative linguistics. Austronesian Studies Relating to Taiwan.

[pgen-0040019-b072] Blust R, Zeitoun E, Li PJ-K (1999). Subgrouping, circularity, and extinction: Some issues in Austronesian comparative linguistics. Selected Papers from the 8th International Conference on Austronesian Linguistics.

[pgen-0040019-b073] Bowcock AM, Ruiz-Linares A, Tomfohrde J, Minch E, Kidd JR (1994). High resolution of human evolutionary trees with polymorphic microsatellites. Nature.

