# Correction: The Genetic Structure of Pacific Islanders

**DOI:** 10.1371/annotation/cbdd11a0-4a29-4e7c-9e4e-c00a184c7777

**Published:** 2008-03-28

**Authors:** Jonathan S Friedlaender, Françoise R Friedlaender, Floyd A Reed, Kenneth K Kidd, Judith R Kidd, Geoffrey K Chambers, Rodney A Lea, Jun-Hun Loo, George Koki, Jason A Hodgson, D. Andrew Merriwether, James L Weber

In the Funding section, what is listed as the National Geographic Society Exploration Fund should be listed as the National Geographic Society.

